# Integrating carbon emission, accumulation and transport in inland waters to understand their role in the global carbon cycle

**DOI:** 10.1111/gcb.15448

**Published:** 2020-12-09

**Authors:** Dominic Vachon, Ryan A. Sponseller, Jan Karlsson

**Affiliations:** ^1^ Climate Impacts Research Centre (CIRC) Department of Ecology and Environmental Science Umeå University Umeå Sweden

**Keywords:** carbon cycle, conceptual framework, coupled fluxes, global change, inland waters, terrestrial carbon fate

## Abstract

Inland waters receive a significant quantity of carbon (C) from land. The fate of this C during transit, whether it is emitted to the atmosphere, accumulated in sediments or transported to the ocean, can considerably reshape the landscape C balance. However, these different fates of terrestrial C are not independent but are instead linked via several catchment and aquatic processes. Thus, according to mass conservation, any environmental change inducing a shift in a particular C fate should come at the expense of at least one other fate. Nonetheless, studies that have investigated C emission, accumulation and transport concertedly are scarce, resulting in fragmented knowledge of the role of inland waters in the global C cycle. Here, we propose a framework to understand how different C fates in aquatic systems are interlinked and covary under environmental changes. First, to explore how C fates are currently distributed in streams, rivers, reservoirs and lakes, we compiled data from the literature and show that ‘C fate allocation’ varies widely both within and among inland water systems types. Secondly, we developed a framework that integrates C fates in any inland water system by identifying the key processes underlying their linkages. Our framework places the partitioning between the different C forms, and how this is controlled by export from land, internal transformations and hydrology, as central to understanding C fate allocation. We argue that, by focusing on a single fate, studies could risk drawing misleading conclusions regarding how environmental changes will alter the role of inland waters in the global C cycle. Our framework thus allows us to holistically assess the consequences of such changes on coupled C fluxes, setting a foundation for understanding the contemporary and future fate of land‐derived C in inland water systems.

## Graphical abstract

The different fates of terrestrial C in inland waters, whether it is emitted, stored or transported downstream, are linked via various catchment and aquatic processes. According to mass conservation, any environmental change inducing a shift in a particular C fate should come at the expense of at least one other fate, thus modifying the ‘C fate allocation’. Here we propose a framework that allows us to holistically assess the consequences of such changes on the coupled C fluxes of inland waters, setting a foundation for understanding the contemporary and future fate of land‐derived C in inland water systems.

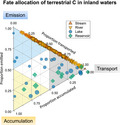

## 1 INTRODUCTION

The millions of streams, rivers, ponds and lakes on Earth cover only about 3% of the land surface, yet the amount of carbon (C) they collectively emit to the atmosphere, accumulate in sediments and transport to the ocean can significantly impact the land C balance (Cole et al., 2007; Le Quéré et al., 2018). By gathering C flux estimates across inland water systems, one can practically assess the proportional fate of terrestrial C at regional (Butman et al., 2016; Stackpoole et al., 2017; Webb et al., 2018) and global scales (Cole et al., 2007; Drake et al., 2018). Although plagued with large uncertainties (Drake et al., 2018), such large‐scale C flux estimates have reinforced the quantitative importance of inland waters in a regional and global context. Nonetheless, even if accurate estimates are finally achieved, these are not static but will instead fluctuate constantly due to ongoing climate and land‐use change (e.g. Maavara et al., 2017; Regnier et al., 2013). Therefore, a more mechanistic understanding of the aquatic C cycle in inland waters is required if we hope to predict the impact of environmental changes on the C balance of land.

**FIGURE 1:**
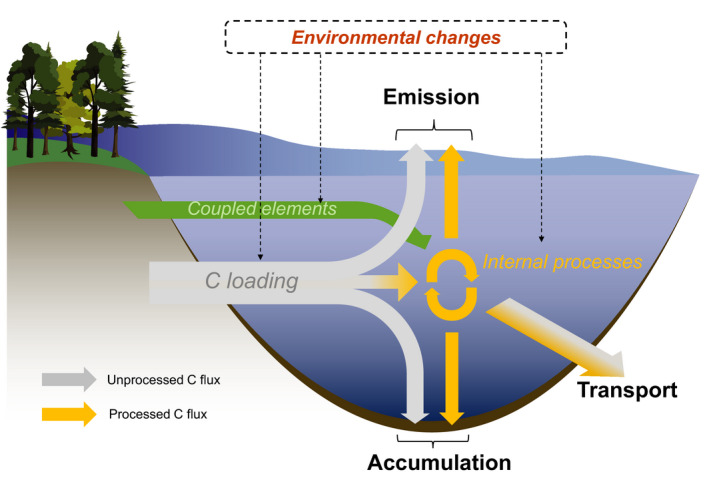
The fates of transiting C in inland waters include emission to the atmosphere, accumulation in sediments and transport to downstream systems. Each fate can occur directly (grey arrows) or after being processed (yellow arrows). Coupled element inputs from the catchment (green arrow) such as nutrients also influence internal C processing and fate. By affecting both catchment and aquatic processes, the impacts of environmental changes such as shifts in climate and catchment disturbances on C fluxes can be complex and require an integrative approach

Many studies have investigated the mechanisms underlying C emissions, accumulation and transport and these efforts are fundamental for understanding how specific processes may respond to changing drivers. However, the different fates of terrestrial C in inland waters are interconnected via internal and catchment processes (Figure 1). Therefore, according to mass conservation, the C gained or lost by a shift in a particular flux should be compensated by adjustment to its complementary flux. Despite this, most studies address these fates independently, and this focus on a single flux (e.g. emissions) in isolation could lead to erroneous understanding and predictions of the dynamic behaviour of the integrated inland water C cycle. In this context, we propose a framework for understanding the role of inland waters in the global C cycle under environmental changes. This framework explicitly integrates C emission, accumulation and transport, and identifies the different aquatic processes that link these fates together. In this way, our framework sets a foundation to achieve a more integrative understanding of the current and future role of inland waters in regional and global C cycles.

## 2 INTEGRATING C EMISSION, ACCUMULATION AND TRANSPORT IN INLAND WATERS

The role of any stream, river, reservoir or lake in the land C cycle is determined by the degree to which transiting C is locally emitted, accumulated in sediments or transported downstream. Specifically, terrestrial C that is emitted and exported from inland waters reduces the landscape C sink efficiency, while terrestrial or atmospheric C that is accumulated in sediments maintains or increases this C sink efficiency (Chapin et al., 2006). Fragmented information on these fluxes can lead to a biased perception of their function. Nonetheless, guided by the notion that flowing waters mainly transport C and that non‐flowing waters retain C, these fluxes are often measured independently. Indeed, inherent differences related to system geomorphology and hydrodynamics may favour a particular C fate at the expense of others. For example, long‐term C accumulation rates are not typically measured in streams and rivers as they are often assumed to be negligible compared to the other fluxes. Yet, C can accumulate in sediments of streams (Jones, 1997; Webster & Meyer, 1997), large rivers (Bertassoli et al., 2017) and river floodplains (Lininger et al., 2018; Wohl & Pfeiffer, 2018), but these rates have never been integrated with emissions and transport. Likewise, C transport from lakes and reservoirs is rarely estimated because the focus has been mostly on C retention due to long water residence times (WRT) that favour emission and accumulation. However, a few lakes and reservoir C budgets suggest that transport can be a significant C flux (Ejarque et al., 2018; Knoll et al., 2013), especially for inorganic C. Overall, by neglecting one or two of the three main C fates, we may miss valuable information about the integrated function of inland waters in the land C cycle.

**FIGURE 2:**
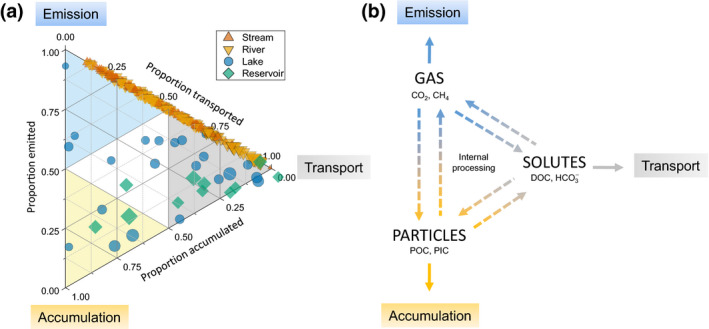
(a) Relative proportion of land C loaded attributed to each fate from 21 different studies (see Supporting Information) comprising C budgets for 22 lakes (blue circles), 11 reservoirs (green diamonds), 72 streams (discharge <1 m^3^ s^−1^; orange triangles) and 87 rivers (discharge >1 m^3^ s^−1^; yellow triangles). Size of the symbol scales with the surface area of the system. The position of each system in the ternary plot determines the dominant fate of the received/transiting C towards emission (upper blue sub‐triangle), accumulation (lower yellow sub‐triangle) and transport downstream (right‐side grey sub‐triangle). Systems falling outside the plot suggest atmospheric inputs of carbon dioxide (CO_2_) (i.e. negative emissions). (b) A conceptual model for determining the fate of C based on the form. Gas leads to emission (or transport; see Box 1), solute leads to downstream transport and particle leads to accumulation (or transport; see Box 1). Aquatic transformation of C between the different forms are represented by the dashed arrows

A survey of the literature reveals a few studies that have reported all three C fluxes together (33 lakes/reservoirs; Figure 2a; Supporting Information). If we assume that C accumulation in streams and rivers is negligible, we can bring in an additional 72 streams and 87 rivers to explore the partitioning of different C fluxes (or ‘fate allocation’) across inland water systems (Figure 2a). While this is a coarse assessment, compiling these data illustrates a wide range in fate allocation within and among system types. For example, streams and rivers encompass almost the entire range between emission and transport, even though some sites could potentially accumulate C, which would result in points departing downwards from the emission‐transport axis line (Figure 2a). By comparison, lakes and reservoirs showed a wider range of flux allocation, covering almost the entire space of this ternary plot (Figure 2a). The different positions in this plot imply fundamental differences between systems related to the nature of the C received, the way C is transformed internally, and the time (i.e. WRT) allowed for transformation. Such large variability in fate allocation, from just a handful of studies, underscores the importance of mechanistically understanding how these C fluxes are coupled and partitioned within and across inland water ecosystems.

**FIGURE 3:**
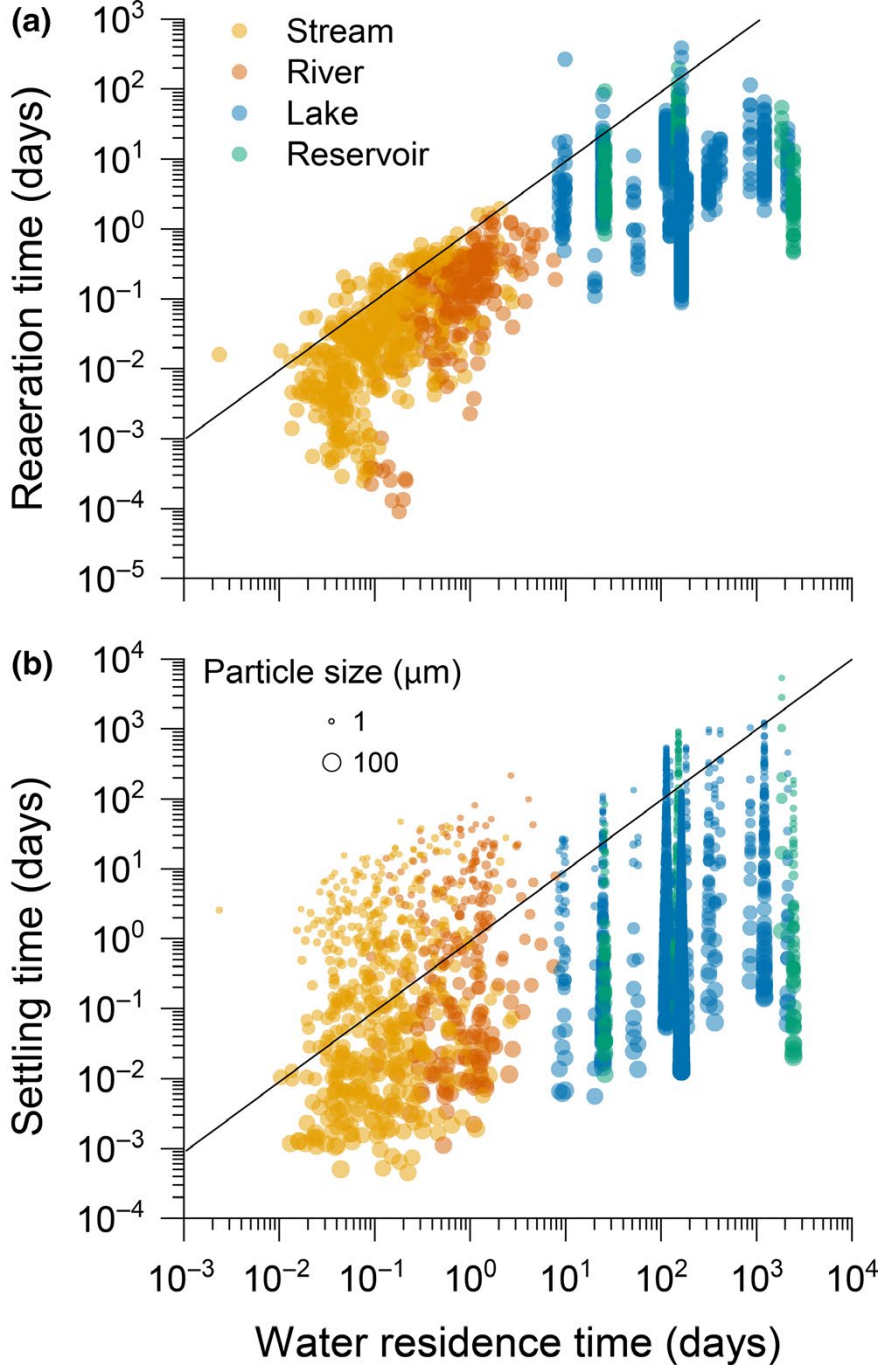
Relationships between water residence time (WRT) and (a) the reaeration time of gas, and (b) settling time of particles, for different system types (streams, rivers, lakes and reservoirs). The reaeration time for gas is the average time required to equilibrate the water column with the atmosphere and is calculated as the average depth of the system (m) divided by the measured gas transfer velocity (m day^−1^). The particle settling time is the average time particles take to sink to the bottom and was calculated as the average system depth (m) divided by the sinking velocity of particles (m day^−1^). The WRT (days), or the average time water takes to leave the system once it has entered, was calculated as the system volume (area (or length × width) × average depth; m^3^) divided by water discharge (m^3^ day^−1^). The size of symbols in (b) is scaled with particle size used in the Stokes' law calculation (see Supporting Information for further details)

Despite the paucity of studies on combined C fluxes (Figure 2), we can generate hypotheses that describe the controls over fate allocation based on current knowledge of aquatic C cycling. First, we suggest that the fate of terrestrial C transiting in an inland water system is ultimately determined by its form (Figure 2b), which is mainly set by catchment properties (Li et al., 2015; Tank et al., 2012). For C to be emitted to the atmosphere, it has to be in the form of gases such as carbon dioxide (CO_2_) and methane (CH_4_). To be settled or physically retained, and accumulated over a longer period, C has to be in the particulate form (e.g. POC, CaCO_3_). To be transported downstream, C can be in any form (see Box 1; Figure 3), although solutes such as dissolved organic carbon (DOC) and bicarbonate (${\text{HCO}}_{3}^{‐}$) should have a higher probability of being transported when compared to particles or gases. Second, driven by coupled element inputs from the catchment and favourable physical conditions, C can also be transformed from one form to another, reshaping the overall fate allocation (Figure 2b). Inland waters can transform all C forms, and indeed C can go through different forms many times before being emitted, transported or accumulated (Battin et al., 2008). This active function of inland waters affects the C fate by chemically and biologically transforming inorganic and organic C during transit (dashed arrows; Figure 2b) via well‐documented processes (e.g. primary production and respiration). Emission, transport and accumulation rates in inland water systems are thus linked, both externally (through catchment processes determining the form of C loaded) and internally (via aquatic transformation), emphasizing the need for more studies that include all fluxes. Importantly, any increase in the rate of one fate should reduce the rate of at least one of the other fates (i.e. moving a point in the ternary plot). In the next section, we explore how we can better understand C fate allocation in inland water systems by focusing on the conditions and factors that determine whether transiting C will be transformed or not.

Box 1 **Physical considerations shaping the fate of gases and particles**

Whether gas is emitted or transported, and a particle is settled or transported can be evaluated from first principles. For example, gases have a higher probability of being transported downstream if the average time it takes for gas to equilibrate with the atmosphere (reaeration time) is slower than the average WRT of a system. Similarly, C particles have a higher probability of being transported if the average time it takes to settle in the sediment is slower than the average WRT of the system.

Here we calculated for a set of streams, rivers, lakes and reservoirs the average time needed for gases to equilibrate with the atmosphere using published estimates of gas transfer velocities (*k*; Klaus & Vachon, 2020; Ulseth et al., 2019) and the average depth of the system (see Supporting Information) compared to the average WRT. In the majority of systems, the average time of atmospheric equilibration is shorter than the WRT (Figure 3a), suggesting that gases have a higher probability of being evaded than transported, regardless of the hydrological regime or system type. In running waters, WRT is much shorter, yet greater turbulence and the reduced depth promote high *k* (Ulseth et al., 2019). Although gas exchanges are slower in lakes and reservoirs (Klaus & Vachon, 2020), their longer WRT allows gas in the water column to be equilibrated with the atmosphere before transiting to downstream systems (Figure 3a). Restricting gas exchange with the atmosphere could, however, promote downstream transport, e.g. as a result of ice cover or permanent stratification in lakes or reservoirs.

The time it takes for a particle to settle in sediments generally depends on its density difference with the water, and its size (i.e. Stokes law). In our assessment, the average sinking velocity appears faster than average WRT in lakes and reservoirs, suggesting that particles have a higher probability to accumulate in such systems (Figure 3b). In running waters, the fate of particles appears far more variable and depends more on particle size. In most systems, though, very fine particles (~1–10 μm spherical diameter) have a higher probability of being transported (Figure 3b). Streams and rivers also show the potential for accumulating larger particles. Once a particle has settled, however, several processes may remove, re‐suspend or mineralize the deposited C. The long‐term storage of C in any system is thus determined by the net balance between deposition and removal (i.e. burial efficiency).

## 3 CARBON TRANSFORMATION IN INLAND WATERS ALTERS THE FATE ALLOCATION

**FIGURE 4:**
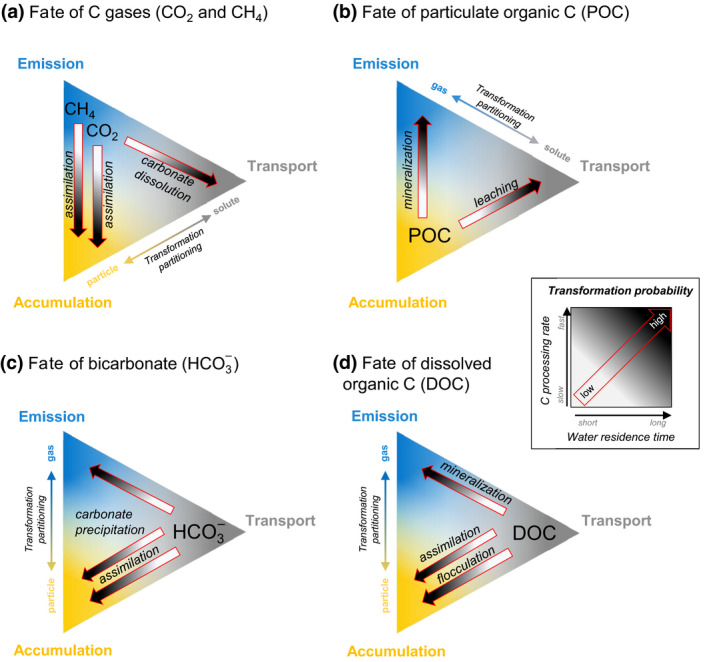
Role of C transformation in the fates of land‐derived (a) carbon dioxide (CO_2_) and methane (CH_4_), (b) particulate organic carbon (POC), (c) bicarbonate (${\text{HCO}}_{3}^{‐}$), and (d) dissolved organic carbon (DOC) transiting in a given inland water system. Coloured areas within each triangle represent the hypothesized probability of each fate: emission (blue), accumulation (yellow) and downstream transport (grey). The fate of each C form depends on the transformation probability of each process (red arrows), which depends on the interaction between the C processing rate and the water residence time (WRT). In general, fast C processing rates and long WRT facilitate transformations, while slow C processing rates and short WRT limit transformations. However, transformation is still possible under short WRT if the C processing is fast, or under slow C processing rate if WRT is long enough. Note that gases and particles can also be transported without prior transformation into solutes (see Box 1). The ‘Transformation partitioning’ arrows show the major direction of the transformation partitioning between different C forms leading to different fates

Starting from the premise that gases should be emitted, particles accumulated and solutes transported (but see Box 1), we developed hypotheses about the conditions under which C can be transformed and fate allocation shifted (Figure 4). These conditions are partly related to energy inputs such as light and temperature, and watershed‐controlled mass inputs such as nutrients (and other elements) and organic C degradability, which all affect the C processing rate. The magnitude of transformation within a given system will also be a function of WRT, as it ultimately determines the time allowed for various processes to influence C fate. For most C species, for example, internal transformation is most probable under long WRT, greater light availability, warmer temperatures, increased nutrient inputs and more degradable organic C. Interestingly, if processing rates are fast enough, transformation can occur and the fate can shift at any WRT. Likewise, C could remain untransformed under long WRT conditions, if processing rates are slow. The transformation from one form to another creates a link between at least two fates. Here we expand on the links between these different fates, based on the C forms involved in the processes.

### 3.1 Transformations between gases and particles link emission and accumulation

The individual fate and the strength of the link between gases and particles rely on the magnitude of the transformation of these forms (Figure 4a,b). If no transformation occurs, the WRT is likely long enough in most inland waters to allow a major part of CO_2_ and CH_4_ inputs to be emitted, and particulate organic carbon (POC; especially the larger particles) to be settled, within ecosystem boundaries (Box 1). Once settled or physically retained, particulate C can be stored for considerably long periods if the conditions prevent transformation (Sobek et al., 2009).

Transformation of CO_2_ or CH_4_ into POC and vice versa links emission and accumulation (vertical dashed arrows; Figure 4a,b). For example, terrestrially derived CO_2_ can be partly assimilated by primary producers instead of being emitted (Hotchkiss et al., 2015; Vachon et al., 2020) and partly be stored in sediments as POC (accumulation; Figure 4a). If primary production is important (e.g. given sufficient light and nutrients) or if terrestrial CO_2_ inputs are limited, then CO_2_ can be provided directly from the atmosphere, resulting in influxes (or ‘negative’ emission). Methane can also be assimilated by microbes/methanotrophs and be incorporated in the aquatic food web (Bastviken et al., 2003), and thus potentially contribute to C accumulation if the resulting biomass is not recycled. Conversely, POC can be converted to gases (Figure 4b) if mineralized in the water column (Attermeyer et al., 2018) or the sediments (Gudasz et al., 2010), thereby completing the link between emission and accumulation.

Gases and particles can also be transformed into solutes and provide a connection between emissions/accumulation and transport (dissolution; Figure 4a,b). For example, CO_2_ can react with inputs of particles of calcium carbonate (CaCO_3_) from the catchment and be dissolved to ${\text{HCO}}_{3}^{‐}$, releasing a calcium ion (Ca^2+^). In this case, the transformation of CO_2_ into a solute results in reduced emission and increased transport (St. Pierre et al., 2019), emphasizing the importance of catchment properties such as bedrock and soil composition. POC can also leach dissolved organic carbon (DOC), which can potentially be transported out of the system (Meyer & O'Hop, 1983). Although observable, these solute‐forming processes are not typically or persistently dominant and are relatively less widespread than the other aforementioned processes.

### 3.2 Solute transformations reduce transport

By definition, C solutes follow water masses and should be transported downstream. However, some fraction of dissolved C species is reactive to biological and chemical processes and can be transformed into gases or particles (Figure 4c,d). Such transformations shift the fate of the loaded solutes from transport towards emission to the atmosphere or accumulation in sediments.

Dissolved inorganic C, such as bicarbonate (${\text{HCO}}_{3}^{‐}$), can react with other inorganic C species, and with calcium (or other cations). Bicarbonate inputs to inland waters can thus be transformed to calcite (CaCO_3_) and CO_2_ (i.e. calcite precipitation; Deemer et al., 2020; Müller et al., 2016; Figure 4c), thereby simultaneously generating CO_2_ emissions and inorganic C accumulation in the sediments (Einsele et al., 2001; Müller et al., 2006; Nõges et al., 2016). Although calcite precipitation can explain a significant proportion of CO_2_ emissions in some lakes and reservoirs with high pH and alkalinity (Khan et al., 2020; Marcé et al., 2015), this still represents a small fraction of the total ${\text{HCO}}_{3}^{‐}$ pool and most of the transiting ${\text{HCO}}_{3}^{‐}$ will be transported to downstream systems (Knoll et al., 2013; Nõges et al., 2016). Bicarbonate can also be assimilated by algae (McConnaughey et al., 1994; Sand‐Jensen et al., 2018) and aquatic plants, especially when CO_2_ is limiting (Iversen et al., 2019).

Dissolved organic carbon molecules are extremely diverse in natural inland waters (Kellerman et al., 2014), resulting in a wide range of degradability. On one hand, a fraction of the DOC pool can be persistent in aquatic networks (Kothawala et al., 2012), which leads to transport and limited mineralization (Wollheim et al., 2015). On the other hand, many studies have shown substantial DOC removal within aquatic networks, either transformed to CO_2_ or POC, and this is often a function of WRT (Algesten et al., 2003; Lupon et al., 2019; Vachon et al., 2017). The degree of DOC degradability, together with variation in ambient conditions like temperature, will further influence the probability of DOC to be mineralized. If the loaded DOC is more biologically or photochemically reactive and if conditions are favourable (warmer temperature for biological mineralization and high UV light for photo‐mineralization, long enough WRT), it should be transformed to gases and be emitted. DOC can also be assimilated by bacteria and contribute to biomass production (Berggren et al., 2010; Guillemette et al., 2013; Meyer et al., 1987), which can partly be accumulated in sediments. If WRT is long but the loaded DOC is less reactive and temperatures are low, there is a higher probability that DOC will form aggregates by flocculation (von Wachenfeldt & Tranvik, 2008) or be adsorbed to mineral surfaces (Groeneveld et al., 2020), and be accumulated in the sediments. The most widespread processes involved in the fate of DOC and ${\text{HCO}}_{3}^{‐}$ in inland waters are thus tightly linking emission, accumulation and transport together.

## 4 IMPLICATIONS AND CONCLUSIONS

The study of inland water C cycling has made tremendous progress in the previous decades (Tranvik et al., 2018), yet this knowledge needs to be integrated to understand their overall role in regional and global C cycles. Here we identify the lack of C flux integration as a potential pitfall in our way towards understanding how inland waters function in the broader C cycle under environmental changes. Although measuring all three fluxes might not be critical for all research questions, and can be logistically challenging, it is in many cases a necessary approach to understand the consequences of environmental change. For example, C loading to inland waters is predicted to be altered (Butman et al., 2015; Raymond et al., 2008; Regnier et al., 2013), yet monitoring C transport only could potentially miss this change if, for example, the loaded C is mainly mineralized and evaded to the atmosphere (Serikova et al., 2018), or accumulated in sediments. Furthermore, the effect of increased nutrient inputs to inland waters due to land‐use change should affect both CO_2_ emission and organic C accumulation simultaneously (Pacheco et al., 2014). However, most studies to date have investigated the impacts of nutrients on one or the other flux separately (e.g. Anderson et al., 2020; Beaulieu et al., 2019). Changes in CO_2_ emission and C accumulation are inversely linked (i.e. this is the same C) and may be potentially accounted for twice. By focusing on only one C flux, we may overlook the counterbalancing effects on the other fluxes.

Environmental changes such as shifts in climate and land‐use exert myriad pressures on inland water functioning, resulting in complex and hard‐to‐predict responses in C fluxes. Here we do not attempt to solve this complexity, but instead, provide a framework that takes into account the potential repercussions of changing one process or flux on other complementary C fluxes. We hope that this framework can guide future studies on the role of inland water systems under environmental change—by advancing a focus on ‘fate allocation’ and by explicitly considering the counterbalancing effects different fates have on each other. Furthermore, the consequences of shifting one C flux at a given time and place may have repercussions later in time or elsewhere within the aquatic network. In these cases, longer time‐ and spatial‐frames might be required to completely ensure the mass conservation principle (e.g. the meta‐ecosystem concept; Loreau et al., 2003). This holistic approach is necessary for our attempt to understand complex spatial and temporal patterns and to ensure a concerted and coherent comprehension of C emission, accumulation and transport in the landscape.

## Supporting information

Supplementary Material

Table S1

## Data Availability

The list of the compiled data that supports the findings of this study is available in the Supporting Information of this article.
