# Supplementary material for: Integrating carbon emission, accumulation and transport in inland waters to understand their role in the global carbon cycle
Source: Glob Chang Biol. 2020 Dec 9;27(4):719–27. doi: 10.1111/gcb.15448 (PMC7898617; doi:10.1111/gcb.15448)
Supplement: Supplementary file 1 — Supplementary Material [file GCB-27-719-s001.docx]

# Supplementary Material for

# Integrating carbon emission, accumulation, and transport in inland waters to understand their role in the global carbon cycle

**Dominic Vachon, Ryan A. Sponseller and Jan Karlsson**

***Climate Impacts Research Centre (CIRC), Department of Ecology and Environmental Science, Umeå University, Umeå, Sweden***

## ****Supplementary Material Content:****

**S1. Literature review of combined C emission, accumulation and transport used in Fig. 2**

**S2. Water residence time, gas reaeration time, and particles settling time calculations**

S3. Supplementary references

## S1. Literature review of combined C emission, accumulation and transport used in Fig. 2

We compiled published data from streams, rivers, lakes, and reservoirs for which C emission, storage (i.e. burial) and transport downstream (or export) were all reported. For streams and rivers, C storage or accumulation are rarely reported, and when this is the case, the other fluxes are not reported. Therefore, we assumed relatively non-significant accumulation, compared to emission and transport. All fluxes were converted to the surface area of the aquatic system, and to annual fluxes. Specifically, these estimates of emission, accumulation, and transport C fluxes come from 33 lakes and reservoirs and 159 streams and rivers (See references in Supp. Table S1). Most of the C emissions reported are in the predominant form of CO_2_, however, CH_4_ was also added when available (Supp. Table S1). Accumulation rates, usually called burial in the publications, are most often for organic carbon, although 5 studies also provided the inorganic accumulation rates (Supp. Table S1).

## S2. Water residence time, gas reareation time, and particles settling time calculations

We estimated water residence time, reareation time, and settling time for a set of streams, rivers, lakes, and reservoirs (Fig. 3 in the main text). The compiled datasets for lakes/reservoirs (Klaus and Vachon 2020), and streams/rivers (Ulseth et al. 2019) originally reported gas transfer velocity (*k*) with additional morphometric characteristics such as volume and mean depth for lakes/reservoirs and discharge and velocity for streams/rivers.

*Water residence time and transit time*

Here we use the water residence time (WRT; the average ‘age’ of water; days) and the water transit time (the time water takes to travel from the entry point to the exit point) as equivalent hydrological metrics. This is true when water enters at one end of the system and exits at the opposite end (Bolin and Rodhe 1973). For lakes, water residence time (WRT) was retrieved from the original publication or, when not available, estimated from an empirical relationship with lake volume (Kalff 2002): ln(WRT) = 0.26 * ln(vol) + 0.55, where WRT is in years and volume in km^3^. For streams and rivers, we estimated WRT as equivalent to the water transit time using water velocity and the system length. We estimated the system length (L; km) from its scaling relationship with discharge (Q) (Burgers et al. 2014). Here the system length (called mainstem length) was operationally defined by an upstream search procedure starting from the discharge monitoring station to the upmost stream by always following the largest catchment flow pathway (see Vörösmarty et al. 2000 for further details).

*Gas reareation time*

Gas transfer velocity (*k*) can be viewed as the depth of water that is equilibrated with the atmosphere per unit of time. Therefore, with the published system mean depth and *k* of the compiled datasets we calculated the gas “reareation time” as:

reareation time (d) = Z_mean_ (m) / k (m d^-1^).

*Particle settling time*

Settling time is the average time the particles take to settle in the sediments: settling time (d) = Z_mean_ (m) / S_vel_ (m d^-1^), where S_vel_ is the settling velocity. We used Stokes’ Law to estimate this as:

S_vel_ as $\frac{\left( \rho_{p}-\rho_{w} \right)gD^{2}}{18\eta}$,

where *D* is the spherical diameter of particles (here we used a range from 1 to 100 μm) and the density of particles ($\rho_{p}$) is assumed to be 1250 kg m^-3^ (organic matter) (Minshall et al. 2000). *η* and $\rho_{w}$ are the kinematic viscosity (0.00113 kg m^-1^ s^-1^) and the density (999.1 kg m^-3^) of water at 15°C, respectively. We obtained settling velocities ranging between about 0.01 to 100 m d^-1^, which largely encompass the range reported in lakes and streams (Stabel 1987; Minshall et al. 2000; Thomas et al. 2001). Overall, the majority of particles are likely to be transported to downstream systems in streams, but less in rivers. In lakes and reservoirs, settling time is faster than transit time, which means deposition.

## S3. Supplementary references

Bolin, B., and H. Rodhe. 1973. A note on the concepts of age distribution and transit time in natural reservoirs. Tellus **1**.

Burgers, H. E. R., A. M. Schipper, A. Jan Hendriks, and others. 2014. Size relationships of water discharge in rivers: Scaling of discharge with catchment area, main-stem length and precipitation. Hydrol. Process. **28**: 5769–5775. doi:10.1002/hyp.10087

Kalff, J. 2002. Limnology: inland water ecosystems, Prentice H.

Klaus, M., and D. Vachon. 2020. Challenges of predicting gas transfer velocity from wind measurements over global lakes. Aquat. Sci. **82**. doi:10.1007/s00027-020-00729-9

Minshall, G. W., S. A. Thomas, J. D. Newbold, M. T. Monaghan, and C. E. Cushing. 2000. Physical factors influencing fine organic particle transport and deposition in streams. J. North Am. Benthol. Soc. **19**: 1–16. doi:10.2307/1468278

Stabel, H. H. 1987. Settling velocity and residence time of particles in Lake Constance. Swiss J. Hydrol. **49**: 284–293. doi:10.1007/BF02538289

Thomas, S. A., J. D. Newbold, M. T. Monaghan, G. W. Minshall, T. Georgian, and C. E. Cushing. 2001. The influence of particle size on seston deposition in streams. Limnol. Oceanogr. **46**: 1415–1424. doi:10.4319/lo.2001.46.6.1415

Ulseth, A. J., R. O. H. Jr, M. B. Canadell, H. L. Madinger, A. Niayifar, and T. J. Battin. 2019. Distinct air–water gas exchange regimes in low- and high-energy streams. Nat. Geosci. **1**. doi:10.1038/s41561-019-0324-8

Vörösmarty, C. J., B. M. Fekete, M. Meybeck, and R. B. Lammers. 2000. Geomorphometric attributes of the global system of rivers at 30-minute spatial resolution. J. Hydrol. **237**: 17–39. doi:10.1016/S0022-1694(00)00282-1
